# Prevalence, seasonal variation, and antibiotic resistance pattern of enteric bacterial pathogens among hospitalized diarrheic children in suburban regions of central Kenya

**DOI:** 10.1186/s41182-016-0038-1

**Published:** 2016-11-29

**Authors:** Mohammad Shah, Cyrus Kathiiko, Akihiro Wada, Erick Odoyo, Martin Bundi, Gabriel Miringu, Sora Guyo, Mohamed Karama, Yoshio Ichinose

**Affiliations:** 1 Kenya Research Station, NUITM-KEMRI Project, Nagasaki University Institute of Tropical Medicine, P.O. Box 19993-00202, Nairobi, Kenya; 2 Centre for Infectious Disease Research in Asia and Africa, Nagasaki University Institute of Tropical Medicine, 1-12-4 Sakamoto, Nagasaki City, 852-8523 Japan; 3 Department of Bacteriology, Nagasaki University Institute of Tropical Medicine, 1-12-4 Sakamoto, Nagasaki City, 852-8523 Japan; 4 Leading Graduate School Program, Nagasaki University Graduate School of Biomedical Sciences, 1-12-4 Sakamoto, Nagasaki City, 852-8523 Japan; 5 Center for Public Health Research, KEMRI, P.O. Box 19993-00202, Nairobi, Kenya

**Keywords:** Diarrhea, *E. coli* pathotypes, Seasonal variation, Antimicrobial susceptibility pattern

## Abstract

**Background:**

The epidemiology of enteric pathogens has not been well studied in Kenya because of wide disparities in health status across the country. Therefore, the present study describes the prevalence of enteropathogenic bacteria, their seasonal variation, and antibiotic resistance profiles among hospitalized diarrheic children in a suburban region of central Kenya.

**Methods:**

Fecal samples were collected between July 2009 and December 2013 from a total of 1410 children younger than 5 years, hospitalized with acute diarrhea in Kiambu County Hospital, Kenya. Conventional culture, biochemical, and molecular methods were conducted to identify causative bacterial pathogens and their virulence factors. Antimicrobial susceptibility tests were performed using E-test strips and VITEK-2 advanced expert system (AES) to evaluate the drug-resistance pattern of the isolates.

**Results:**

Of the 1410 isolates, bacterial infections were identified in 474 cases. Diarrheagenic *Escherichia coli* (DEC) was the most frequently isolated pathogen (86.5%). Other pathogens such as *Aeromonas* (5.5%), *Shigella* (4%), *Salmonella* (3.4%), *Providencia* (3.2%), *Vibrio* spp. (1.1%), *Yersinia enterocolitica* (1.1%), and *Plesiomonas shigelloides* (0.2%) were also identified. Mixed bacterial infection was observed among 11.1% of the cases. The highest infection rate was found during the dry season (59.3%, *p* = 0.04). Most of the DEC was found to be multidrug resistant to trimethoprim/sulfamethoxazole 97.6%, amoxicillin 97.6%, erythromycin 96.9%, ampicillin 96.6%, and streptomycin 89%.

**Conclusions:**

This study suggests that DEC is the leading diarrhea-causing bacterial pathogen circulating in central Kenya, and seasonality has a significant effect on its transmission. Proper antibiotic prescription and susceptibility testing is important to guide appropriate antimicrobial therapy.

## Background

Despite significant advances in the understanding of pathogenesis and management, globally acute diarrhea remains the second leading cause of death in children under 5 years of age [1]. It is estimated that globally, 800,000 children under the age of 5 years die annually, mostly in sub-Saharan Africa and South Asia [2]. Currently, the risk of diarrheal diseases has increased fivefold in sub-Saharan Africa as compared to industrialized countries [3]. In sub-Saharan Africa including Kenya, urban migration is on the rise, resulting in rapid expansion of cities. Creation of overcrowding in urban slums, low socioeconomic conditions, poverty, illiteracy, inadequate safe drinking water, poor sanitation, and malnutrition have raised the occurrence of infectious diseases including diarrhea [4].

The major causes of diarrhea differ significantly in developed and developing countries. In the developed countries, diarrhea is seen in the winter with viruses being the primary agents. In the developing world, diarrhea remains a wet season disease with bacteria playing a greater role [5], although rotavirus has been found to be a single dominant enteric pathogen among children in most of the developed and developing countries [6]. Newly virulent enteric pathogens are emerging throughout the world including Kenya. For example, a multidrug-resistant enteroaggregative *Escherichia coli* (*E. coli*) O44, which is associated with acute and persistent diarrhea, has been reported in Kenya [7]. Recently, *Providencia alcalifaciens* was published for the first time as the etiological cause of diarrhea outbreaks in Kiambu [8], and *E. coli* O157 as a cause of significant outbreaks in Swaziland [9]. However, there is limited information on the surveillance of diarrheagenic pathogens and their antimicrobial resistance pattern. As a result, prevention, active intervention, and response are not well defined at the regional or national level.

The aim of this study was to identify the bacterial pathogens associated with diarrhea, its seasonal burden, and antimicrobial resistance patterns among hospitalized children in a suburban region of central Kenya.

## Methods

### Study area

This study was conducted between July 2009 and December 2013 at the Kiambu County Hospital (KCH). Kiambu County is located in the central highlands of Kenya in the former central province, close to Kenya’s capital, Nairobi, and has a population of 1,782,083. It has a 40% rural and 60% urban population owing to Nairobi’s consistent growth. KCH is a government referral hospital with a 316-bed capacity that offers a full range of services to people living mostly in the central region of the country. The rainfall pattern in Kenya is seasonally bimodal, with a long rainy season starting from March to May, while a shorter rainy season lasts from October to November.

### Study population and specimen collection

A total of 1416 children aged 0–60 months who were hospitalized in the pediatric unit of KCH, with complaints of diarrhea (with or without fever or other accompanying symptoms) and had not taken any antimicrobial agent in the preceding week were recruited into this study. Diarrhea was defined as the passage of three or more watery, loose stools in the previous 24 h, as noted by the mother or the household caretaker. Fecal specimens were obtained from stools/rectal swabs and transported to the laboratory at the Centre for Microbiology Research in sterilized container/Cary-Blair transport medium (Liofilchem, Roseto degli Abruzzi, Italy) and processed within 24 h. Specimens obtained in the evening or at night were kept in a refrigerator at 2–8 °C and transported in a cool box the following day. The hospital staff completed a questionnaire for each patient containing the following information: age, sex, clinical symptoms (fever, vomiting, dehydration, etc.), and medical care sought before hospitalization.

### Isolation and identification of pathogenic bacteria

All fecal specimens were cultured on xylose lysine deoxycholate (XLD) agar (Oxoid Ltd., Basingstoke, Hampshire, UK), deoxycholate hydrogen sulfide lactose (DHL) agar, bromothymol blue (BTB) agar, *Salmonella*-*Shigella* (SS) agar, thiosulfate-citrate-bile salt-sucrose (TCBS) agar (Eiken Chemical Company Ltd. Tochigi, Japan), and *Campylobacter* selective Butzler agar (Oxoid Ltd., Basingstoke, Hampshire, UK). In addition to these media, two types of enrichment media were used: alkaline peptone water and selenite broth (HiMedia Laboratories Pvt. Ltd., Mumbai, India). The plates and the broth with the exception of butzler were incubated overnight at 37 °C. Butzler plates were incubated under a micro-aerophilic condition with an Anaeropack® system (Mitsubishi Gas Chemical Company, Inc. Tokyo, Japan) at 42 °C for 48 h. Bacterial isolates were further confirmed by both routine biochemical method [10] and VITEK-2 automated analyzer (bioMérieux, Inc., Durham, NC, USA) system. For each subject, five lactose fermenting and two lactose non-fermenting colonies were subjected to direct PCR for *E. coli* virulence factor testing.

### Serological identification

Serologic identification was performed by the slide agglutination technique with polyvalent and monovalent antisera for serotype identification of *Vibrio cholerae*, *Salmonella*, and *Shigella* spp*.* (Denka Seiken Co., LTD, Japan). Positive agglutination confirmed the serotype and subtype of the isolates. *E. coli* strain ATCC 25922 was used as a negative control.

### Molecular examination

For genetic analysis, bacterial genomic DNA was extracted by the QIAGEN DNA extraction kit (QIAGEN, Hilden, Germany). Multiplex PCR was performed using published primers specific for genes targeting *eae* for enteropathogenic *Escherichia coli* (EPEC); *eae* and *stx* for enterohemorrhagic *Escherichia coli* (EHEC); *est* and *elt* for enterotoxigenic *Escherichia coli* (ETEC); *ipaH* for enteroinvasive *Escherichia coli* (EIEC) and *Shigella* spp.; *aggR*, CVD432, and *aspU* for enteroaggregative *Escherichia coli* (EAEC) [11]; *hisJ* for *Salmonella* spp. [12]; and *toxR* (toxR-F: 5′-TTAACGCTGAATTACATTCA-3′ and toxR-R: 5′-TTAAGATTTACTGAACAGTA-3′) and *ctxA* (ctxA-F: 5′-TCAATTAGTTTGAGAAGTGC-3′ and ctxA-R: 5′-TCAGATTGATAGCCTGAAAA-3′) for *Vibrio* spp. The PCR mixture was prepared with puReTaq Ready-To-Go PCR beads kit (GE Healthcare, UK) according to the manufacturer’s instructions.

### Antimicrobial susceptibility tests

Antimicrobial susceptibility testing of all the 290 randomly selected isolates was performed by E-test (Liofilchem, Roseto degli Abruzzi, Italy) strips and the VITEK-2 advanced expert system (AES) using AST-GN83 cards (bioMérieux, Inc., Durham, NC, USA); minimal inhibitory concentrations (MICs) were determined according to the manufacturer’s instruction manual. The breakpoints used were those recommended by the Clinical and Laboratory Standard Institute [13]. Standard *E. coli* ATCC 25922 of known susceptibility was used as a control organism. Antibiotics tested included amoxicillin, ampicillin, ampicillin/sulbactam, amoxicillin/clavulanate, cefazolin, cefotaxime, cefoxitin, cefuroxime, cefuroxime/axetil, ceftriaxone, ceftazidime, cefepime, meropenem, aztreonam, erythromycin, doxycycline, tetracycline, ciprofloxacin, ofloxacin, chloramphenicol, amikacin, gentamicin, kanamycin, streptomycin, trimethoprim/sulfamethoxazole, and nitrofurantoin.

### Statistical analysis

Frequencies and percentages were calculated for the study variables. Comparisons were drawn using a two-tailed Chi-square test. A *p* value of less than 0.05 was considered to be statistically significant.

## Results

### Characteristics of the study population

During the period between July 2009 and December 2013, a total of 1416 fecal samples were obtained from children hospitalized in KCH with acute diarrhea accompanied by vomiting (64.7%), dehydration (40.6%), and fever (28.7%) for rotavirus pre-vaccination surveillance study (Table 1). Six cases were excluded for missing age data. Remaining 1410 (773, 54.8% males and 637, 45.2% females) fecal samples were tested only for rotavirus and enteric bacterial pathogens. At least one enteric bacterial pathogen was detected in 318 (22.6%) diarrheal cases, and mixed pathogens (pathogenic bacteria/rotavirus) were found in 156 (11.1%) cases. Mixed bacterial infections were seen in association with rotaviruses (138 cases) except in 18 cases where two bacterial copathogens were identified (data not shown).

**Table 1:** Age distribution, clinical symptoms, and other characteristics of hospitalized children with diarrhea

Age distribution, clinical and other characteristics	Number (%)	
	Total case	Bacterial case
Age (months)		
0–6	295 (20.5)	86 (18.1)
7–12	653 (46.3)	226 (47.7)
13–18	238 (16.9)	90 (19.0)
19–24	114 (8.1)	36 (7.6)
25–36	48 (3.4)	18 (3.8)
37–60	62 (4.4)	18 (3.8)
Gender		
Male	773 (54.8)	257 (54.2)
Female	637 (45.2)	217 (45.8)
Season		
Dry	798 (56.6)	281 (59.3)
Rainy	612 (43.4)	193 (40.7)
Clinical symptoms		
Acute diarrhea	1410 (100)	474 (100)
Watery and mucoid stools	1316 (93.3)	391 (82.5)
Bloody stools	94 (6.7)	83 (17.5)
Vomiting	912 (64.7)	402 (84.8)
Severe dehydration	572 (40.6)	226 (47.7)
Fever	405 (28.7)	183 (38.6)

All patients were divided into six age groups (Table 1) with age ranging from 1 month to 5 years, and the highest proportion of bacterial infections (84.8%, 402/474) were observed within 0–18 months old, particularly within the age group of 7–12 months (47.7%). A similar result was also observed among the total number of diarrheal cases (84.1%, 1186/1410). This value was progressively decreased after 18 months.

### Prevalence of pathogenic bacteria and seasonal variation

A wide range of bacterial pathogen was detected in 474 cases. Of these, diarrheagenic *E. coli* (DEC) (86.5%, 410/474) was found to be the most common enteropathogenic bacteria representing EAEC, ETEC, EPEC, EIEC, and EHEC. The distribution of different enteric bacterial pathogen is shown in Table 2. Of the DEC, EAEC (50%, 237/474) was the most prevalent of all bacterial cases of diarrhea, followed by ETEC (24.3%, 115/474), EPEC (11.6%, 55/474), EIEC (0.4%, 2/474), and EHEC (0.2%, 1/474). Among 237 isolated EAEC strains, 105 strains carried both *aspU* and *aggR* genes, 45 strains were found to be positive for *aspU*, *aggR*, and *pCVD432* genes, while those that harbored *aspU* and *aggR* gene alone were 76 and 11 strains, respectively. Of the 115 ETEC strains, heat-stable toxin (ST) and heat-labile toxin (LT) genes were detected in 60 and 41 strains, respectively, and both ST and LT genes were found positive in 14 isolates. A total of 55 EPEC strains were positive for *eae* gene, while *ipaH* gene was found in all four EIEC strains and EHEC strain was positive for both *eae* and *stx* genes.

**Table 2:** Prevalence and seasonal variation of different enteropathogenic bacteria identified in children hospitalized with acute diarrhea

Enteropathogenic bacteria	Number of isolates (%)	Dry season number (%)	Rainy season number (%)	*p* value
Diarrheagenic *E. coli*				
EAEC	237 (50.0)	143 (30.2)	94 (19.8)	0.002
ETEC	115 (24.3)	75 (15.8)	40 (8.4)	0.125
EPEC	55 (11.6)	24 (5.1)	31 (6.5)	0.297
EIEC	2 (0.4)	0	2 (0.4)	N.A.
EHEC	1 (0.2)	0	1 (0.2)	N.A.
*Aeromonas* spp.	26 (5.5)	14 (3.0)	12 (2.5)	0.740
*Shigella* spp.	19 (4.0)	12 (2.5)	7 (1.5)	0.043
*Salmonella* spp.	16 (3.4)	9 (1.9)	7 (1.5)	0.155
*Providencia* spp.	15 (3.2)	11 (2.3)	4 (0.8)	0.738
*Vibrio* spp.	5 (1.1)	5 (1.1)	0	N.A.
*Yersinia enterocolitica*	5 (1.1)	3 (0.6)	2 (0.4)	0.329
*Plesiomonas shigelloides*	1 (0.2)	0	1 (0.2)	N.A.

*N.A.* not applicable

Other pathogens included *Aeromonas* (5.5%, 26/474), *Shigella* (4%, 19/474), *Salmonella* (3.4%, 16/474), *Providencia* spp. (3.2%, 15/474), *Vibrio* spp. (1.1%, 5/474), *Yersinia enterocolitica* (1.1%, 5/474), and *Plesiomonas shigelloides* (0.2%, 1/474). Among *Shigella*, *S. flexneri* (57.9%, 11/19), *S. sonnei* (26.3%, 5/19), *S. boydii* (10.5%, 2/19), and *S. dysenteriae* (5.3%, 1/19) were identified. Three of the five cases of *Vibrio* species were *V. cholerae* O1, and two were *V. fluvialis* confirmed by biochemical test and PCR amplification. There were 16 serotypes of *Salmonella enterica* strains including enteritidis (*n* = 14) and typhimurium (*n* = 2) serotypes. *Campylobacter* spp. was not found during the study period.

Seasonal variation in the occurrence of diarrheal infection was reported. It was observed that a significantly higher percentage (56.6%, 798/1410) of diarrheal cases were detected during the dry season (Dec–Feb and June–Sep) (*p* = 0.001). Also, the prevalence of bacterial infection was found to be higher in the dry season, 59.3% (281/474) than in the rainy season, 40.7% (193/474) (*p* = 0.04). A higher proportion of EAEC (60.3%; *p* = 0.002) and ETEC infection (65.2%; *p* = 0.124) was observed more frequently during the dry season. Relatively high prevalence of EPEC (56.4%; *p* = 0.297) infection occurred during the rainy season (Oct–Nov and Mar–May), but this difference was not statistically significant. Moreover, two EIEC and one EHEC strains were detected during the rainy season. *Salmonella* strains were isolated at almost similar frequencies in both seasons, but a higher rate of *Shigella* spp. (63.2%; *p* = 0.043) were detected during the dry season (Table 2).

### Antimicrobial resistance

Table 3 summarizes the antimicrobial resistance pattern of different bacterial pathogens. Lower resistance rate was observed in carbapenems (3.4%), followed by quinolone (4.8%) and cephalosporin antibiotic groups (6.2–23.5%). Higher resistance was seen in penicillin groups such as amoxicillin (97.2%) and ampicillin (96.6%), followed by macrolide groups such as erythromycin (96.9%), and sulfonamides group such as trimethoprim/sulfamethoxazole (97.6%). Among these, most of the bacterial pathogens showed multidrug-resistant patterns.

**Table 3:** Drug susceptibility patterns of the diarrheagenic *E. coli* (DEC)

Antibiotic class	Antibiotic tested	Susceptibility pattern					
		Sensitive		Intermediate		Resistant	
		*N*	%	*N*	%	*N*	%
Penicillins	Amoxicillin	6	2.1	2	0.7	282	97.2
	Ampicillin	8	2.8	2	0.7	280	96.6
	Ampicillin/sulbactam	46	15.9	50	17.2	194	66.9
	Amoxicillin/clavulanate	143	49.3	79	27.2	68	23.4
Cephalosporin	Cefazolin	218	75.2	4	1.4	68	23.4
	Cefoxitin	263	90.7	9	3.1	18	6.2
	Cefuroxime	219	75.5	4	1.4	67	23.1
	Cefuroxime/axetii	210	72.4	14	4.8	66	22.8
	Cefotaxime	231	79.7	1	0.3	58	20.0
	Ceftriaxone	231	79.7	0	0.0	59	20.3
	Ceftazidime	231	79.7	0	0.0	59	20.3
	Cefepime	231	79.7	0	0.0	59	20.3
Carbapenems	Meropenem	279	96.2	1	0.3	10	3.4
Monobactams	Aztreonam	230	79.3	0	0.0	60	20.7
Macrolides	Erythromycin	0	0.0	9	3.1	281	96.9
Tetracyclines	Doxycycline	28	9.7	140	48.3	122	42.1
	Tetracycline	28	9.7	83	28.6	179	61.7
Quinolone	Ciprofloxacin	275	94.8	1	0.3	14	4.8
	Ofloxacin	275	94.8	1	0.3	14	4.8
Chloramphenicol	Chloramphenicol	200	69.0	23	7.9	67	23.1
Aminoglycosides	Amikacin	281	96.9	0	0.0	9	3.1
	Gentamicin	235	81.0	5	1.7	50	17.2
	Kanamycin	274	94.5	3	1.0	13	4.5
	Streptomycin	32	11.0	0	0.0	258	89.0
Sulfonamides	Trimethoprim/sulfamethoxazole (SXT)	7.0	2.4	0.0	0.0	283	97.6
Nitrofurans	Nitrofurantoin	192	66.2	74	25.5	24	8.3

## Discussion

Diarrheal diseases are a major health concern for children in Kenya as well as other countries in sub-Saharan Africa. Knowledge of the circulating enteropathogen is essential for the implementation of appropriate public health measures to control diarrheal diseases. Currently, Global Enteric Multicenter Study (GEMS) is conducting a comprehensive study of childhood diarrheal diseases at seven sites in Africa and Asia, including western Kenya [14]. As a part of the rotavirus pre-vaccination surveillance study conducted in the suburban region of central Kenya, this is the first report investigating the prevalence, seasonal variation, and the antimicrobial resistance patterns of enteric bacterial pathogens, isolated from the hospitalized diarrheal children younger than 5 years old.

In our study, diarrheal infection was higher among the children in age groups within 0–18 months. This could be due to their underdeveloped immune system that is incapable of mounting an effective immunological response [15]. Also, risk of placing contaminated fingers and fomites in the mouth is greatly increased due to physiological phenomenon like teething and crawling which begins at this age [16]. On the other hand, less diarrheal infection was observed with increasing age groups which is consistent with previous studies [17]. This meant that poor immunity among children invariably due to under nutrition increases susceptibility to diarrheal diseases.

In this study, DEC was the predominant pathogen (86.5%, 410/474) identified in the screened fecal samples. We amplified eight targeted genes by PCR to identify five types of DEC. Among the strains of DEC isolates, EAEC (50%) was the most frequently identified potential pathogen followed by ETEC (24.3%), EPEC (11.6%), EIEC (0.4%), and EHEC (0.2%). The relative frequency of these categories of DEC was similar to that observed in Nigeria [18], Tanzania [19], and Mozambique [20]. Contrary to our findings, a study previously conducted in Kenya [21] and Ghana [22] showed that EPEC was the most frequently identified bacterial pathogen. This could have been due to the divergence of DEC pathotype, since it varies globally from region to region, and even between and within countries in an identical geographic location [23].

The *aggR* gene is a transcriptional activator gene required for the expression of the anti-aggregation protein (dispersin) gene *aap* (also called *aspU*) and an anti-aggregation protein transporter gene *aatA* (also known as CVD432 or AA prove) which have been commonly used for the detection of EAEC [11]. Furthermore, pathogenic studies have suggested that *aggR* gene controls the virulence functions in EAEC [24]. Therefore, strains expressing the *aggR* regulon are denoted as typical EAEC [25]. In most epidemiological studies on EAEC, typical EAEC strain has been mainly targeted because many investigators have found a strong association between these and diarrhea [19, 26, 27], which is consistent with our study. ETEC was reported as the second most common DEC isolated in this study. It is well known that ETEC strains cause diarrhea through the action of the enterotoxin LT and ST. Our findings showed a higher prevalence of ST- than LT-producing ETEC, which was also observed in previous studies [28]. Additionally, *eae* and the *ipaH* genes were used to detect EPEC and EIEC pathotypes, respectively, while both *eae* and *stx* genes were for EHEC [11].

*Shigella*, *Salmonella*, and *Campylobacter* spp. are the well-known pathogens that cause bacterial gastroenteritis in the world. Surprisingly, in the present study, an infrequent number of *Shigella* (4%) and *Salmonella* spp*.* (3.4%) were isolated than in a previously reported study carried out between May 1997 and April 1998 in western Kenya, which showed higher percentages of *Shigella* (44%), *Campylobacter (*30%), and *Salmonella* (14%) infection [29]. In a more recent study carried out in four provinces of Kenya, the isolation rate of *Shigella* (2.3%) and *Salmonella* (3.5%) were found similar to our study [21]. This highlights the declining proportion of diarrhea due to a probable improvement of water supply and sanitation in most parts of the country. Among bacterial pathogens, we also detected *Aeromonas* and *Providencia* spp., and *Plesiomonas shigelloides* which had not been reported previously in this study area.

Mixed infections were observed among 11.1% patients, and most of the cases, bacterial infections were associated with rotaviruses (data not shown). In developing countries, it is not uncommon to isolate more than one enteric pathogen from the same patient. The prevalence of co-infection in our patients is less than in Madagascar (22.8%) [30], but higher than in Egypt (5.9%) [31]. Therefore, the interpretation of data on mixed infection is complicated because not all the potential enteropathogens detected necessarily contribute to the etiology of the patient’s diarrheal disease. Due to the limitation in the study design, we were not able to analyze indicators of severe diarrhea in patients with co-infections that would have prompted a more extensive investigation.

Our results also brought out the effect of seasonal variation on the occurrence of diarrheal pathogens in the environment. A higher infection rate (56.6%) was observed during the dry seasons as compared to the rainy seasons (43.4%), which is in agreement with previous studies [28, 32]. During the dry seasons, shortage of water, high temperature, and dusty conditions are favorable to the proliferation of infectious agents [32]. The highest infection rate of diarrhea caused by ETEC (75/115, 65.2%), EAEC (143/237, 60.3%), and *Shigella* (12/19, 63.2%) was observed more frequently during the dry season while infection caused by EPEC (31/55, 56.4%) was observed during the rainy season. These findings are in agreement with the study conducted in Tanzania except that EPEC was isolated at similar frequencies in both seasons [28]. In contrast, a study in Bangladesh found a higher prevalence of ETEC and EPEC during the dry season [33]. However, it is documented that distinct seasonal patterns of diarrhea occur in many geographical areas. In a temperate climate, bacterial diarrhea occurred more frequently during the warm season, whereas viral diarrhea, particularly diarrhea caused by rotavirus, peaked during the winter. In tropical areas, rotavirus diarrhea occurred throughout the year, increasing in frequency during the drier, cold months, whereas bacterial diarrheas peaked during the warmer, rainy seasons [34].

In this study, 26 antibiotics were tested against randomly selected 290 DEC pathogens (Table 3). Most tested isolates showed more than 90% sensitivity to ciprofloxacin, ofloxacin, amikacin, kanamycin, and cefoxitin probably due to their seldom use [29]. On the contrary, a high proportion of amoxicillin (97.2%), ampicillin (96.6%), erythromycin (96.9%), trimethoprim/sulfamethoxazole (97.6%), streptomycin (89%), ampicillin/sulbactam (67%), and tetracycline (61.7%) resistance was observed. The high rate of resistance towards these antibiotics may be due to their indiscriminate use because they are readily available over the counter [29]. Furthermore, the majority of the *E. coli* isolates in our study displayed resistance to one or more antibiotics including amoxicillin, ampicillin, trimethoprim/sulfamethoxazole, erythromycin, and streptomycin, which was also observed in previous studies in Kenya [7].

## Conclusions

The present study describes the burden of enteric bacterial pathogens in suburban settings of central Kenya. It highlights the involvement of EAEC diarrheal cases in Kiambu regions in a significant proportion and that EAEC may be an emerging pathogen. A high seasonal variation of bacterial infections is also reported. This study further provides updated information on the prevalence of other enteropathogens that have been circulating in this region, which can be useful in setting suitable health intervention strategies or informing future epidemiological studies on diarrheal pathogens in Kenya. The emergence of multidrug-resistant bacteria raises a broader health issue. Therefore, antibiotic treatment should be strictly controlled by the health authority. Continuous monitoring of the resistance pattern is mandatory for the appropriate selection of antimicrobial drugs.

## Abbreviations

BTB: Bromothymol blue
CLSI: Clinical and Laboratory Standard Institute
DEC: Diarrheagenic *E. coli*
DHL: Deoxycholate Hydrogen Sulfide Lactose
EAEC: Enteroaggregative *Escherichia coli*
EHEC: Enterohemorrhagic *Escherichia coli*
EIEC: Enteroinvasive *Escherichia coli*
ELISA: Enzyme-linked immunosorbent assay
EPEC: Enteropathogenic *Escherichia coli*
ETEC: Enterotoxigenic *Escherichia coli*
GEMS: Global Enteric Multicenter Study
KCH: Kiambu County Hospital
PCR: Polymerase chain reaction
SS: *Salmonella*-*Shigella*
TCBS: Thiosulfate-citrate-bile salt sucrose
XLD: Xylose lysine deoxycholate
